# Combined metformin-associated lactic acidosis and euglycemic ketoacidosis

**DOI:** 10.1007/s00508-017-1251-6

**Published:** 2017-09-01

**Authors:** Verena Schwetz, Florian Eisner, Gernot Schilcher, Kathrin Eller, Johannes Plank, Alice Lind, Thomas R. Pieber, Julia K. Mader, Philipp Eller

**Affiliations:** 1 Department of Internal Medicine, Division of Endocrinology and Diabetology, Medical University of Graz, Auenbruggerplatz 15, 8036 Graz, Austria; 2 Department of Internal Medicine, Intensive Care Unit, Medical University of Graz, Graz, Austria; 3 Division of Nephrology, Department of Internal Medicine, Graz, Austria; 4 Division of Gastroenterology and Hepatology, Department of Internal Medicine, Graz, Austria

**Keywords:** Ketoacidosis, Type 2 diabetes, Metformin, Lactic acidosis

## Abstract

**Background:**

In renal failure metformin can lead to lactic acidosis. Additional inhibition of hepatic gluconeogenesis by accumulation of the drug may aggravate fasting-induced ketoacidosis. We report the occurrence of metformin-associated lactic acidosis (MALA) with concurrent euglycemic ketoacidosis (MALKA) in three patients with renal failure.

**Case presentations:**

Patient 1: a 78-year-old woman (pH = 6.89, lactic acid 22 mmol/l, serum ketoacids 7.4 mmol/l and blood glucose 63 mg/dl) on metformin and insulin treatment. Patient 2: a 79-year-old woman on metformin treatment (pH = 6.80, lactic acid 14.7 mmol/l, serum ketoacids 6.4 mmol/l and blood glucose 76 mg/dl). Patient 3: a 71-year-old man on metformin, canagliflozin and liraglutide treatment (pH = 7.21, lactic acid 5.9 mmol/l, serum ketoacids 16 mmol/l and blood glucose 150 mg/dl). In all patients, ketoacidosis receded on glucose infusion and renal replacement therapy.

**Conclusion:**

This case series highlights the parallel occurrence of MALA and euglycemic ketoacidosis, the latter exceeding ketosis due to starvation, suggesting a metformin-triggered inhibition of gluconeogenesis. Affected patients benefit from glucose infusion counteracting suppressed hepatic gluconeogenesis.

## Background

Metformin is the most commonly used oral antihyperglycemic drug in type 2 diabetes. Metformin-associated lactic acidosis (MALA) is a rare but severe adverse effect in patients with renal failure. Besides elevation of lactate, acidosis has been attributed to uremia. In severe cases hemodialysis is recommended. Metformin increases the activity of AMP-dependent protein kinase, stimulating fatty acid oxidation, glucose uptake, non-oxidative metabolism, and reducing lipogenesis and gluconeogenesis [1], resulting in diminished hepatic glucose production and lower blood glucose levels. In an experimental setting, administration of metformin increased ketoacid production [2]. In renal failure and MALA, accumulation of metformin may lead to increased ketogenesis adding to the acid disturbance.

In this article we report three cases of metformin-associated lactic acidosis and euglycemic ketoacidosis (MALKA), diabetes and acute renal failure identified in 2015 at the intensive care unit (ICU) of a tertiary center. Serum beta-hydroxybutyrate levels (0.0–8.0 mmol/l) were measured by Precision Xtra Blood β‑Ketone Test Strips (Abbott Diabetes Care, Witney, UK).

## Case series

**Fig. 1:**
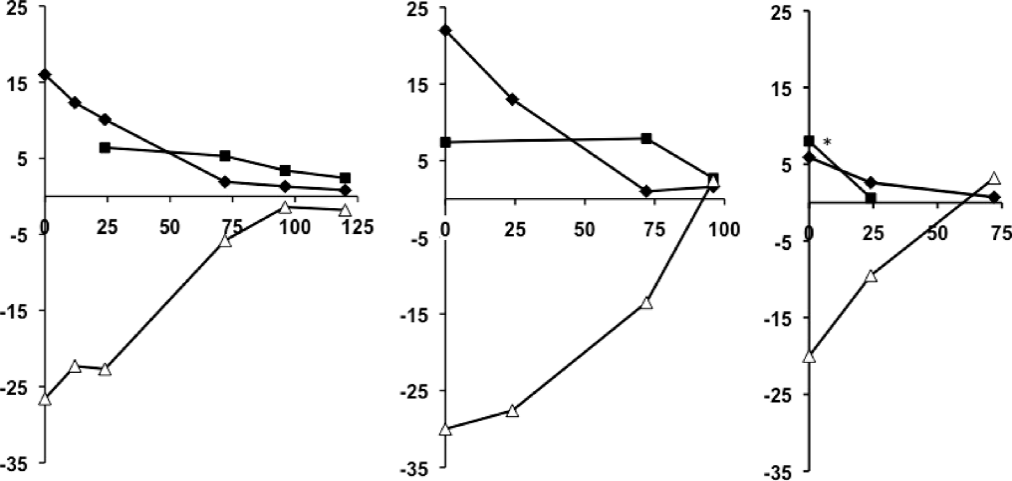
Metformin-associated lactic acidosis and euglycemic ketoacidosis in three critically ill patients. Blood gas analyses with serum levels of lactic acid (*closed diamonds*), ketoacids (0.00–8.00 mmol/l; *closed squares*), and base excess levels (*open triangles*) are plotted for patient 1 (**a**), patient 2 (**b**), and patient 3 (**c**) as a function of time (h) on the abscissa. * Ketoacid levels exceeded 8 mmol/l in this patient, the true value was beyond the range of measurement

Patient 1: a 78-year-old woman with a body mass index (BMI) of 26.3 kg/m^2^ on metformin and insulin therapy with chronic kidney disease (CKD) stage IIIa due to traumatic unilateral nephrectomy was admitted to the emergency department (ED) with nausea, vomiting, acute on chronic renal failure (creatinine 796.5 µmol/l, blood urea nitrogen 28.4 mmol/l) and metabolic acidosis with Kussmaul breathing (pH = 6.89, HCO_3_ = 5 mmol/l, pCO_2_ = 17.3 mm Hg or 2.3 kPa). The premedication included metformin 850 mg b.i. d. and biphasic insulin aspart b.i. d. (16–0–12 IU). On admission to the ICU, the patient was somnolent, the blood pressure (BP) was 103/45 mm Hg, body temperature 34.1 °C, oxygen saturation 97%, heart rate (HR) 76/min, arterial pO_2_ 132 mm Hg (=17.6 kPa), base excess −30 mmol/l, anion gap 47 mmol/l and serum lactic acid level 22 mmol/l. Moreover, the patient displayed elevated serum ketoacids of 7.4 mmol/l and blood glucose (BG) levels of 63 mg/dl. Laboratory findings were as follows: C‑peptide 0.8 ng/ml (0.78–1.89), tyrosine phosphatase antibodies 0.0 U/ml (0.0–20.0), glutamic acid decarboxylase antibodies 0.3 U/l (0.0–9.5). Cranial computed tomography scan and chest X‑ray showed no pathologies. Initial treatment included hemodialysis, bicarbonate substitution, and glucose infusion. The high anion gap acidosis slowly receded during the following days (Fig. 1a). After initial stabilization, the patient acquired pneumonia requiring invasive mechanical ventilation and ongoing renal replacement therapy but finally passed away.

Patient 2: a 79-year-old woman (BMI 28.4 kg/m^2^) on metformin therapy with previous CKD IIIb on metformin 1000 mg b.i. d., candesartan, gabapentin, phenprocoumon, and pantoprazol was admitted to our ED. She had gastroenteritis with acute on chronic renal failure (creatinine: 796.5 µmol/l, blood urea nitrogen: 27.6 mmol/l), overt hyperkalemia of 7.7 mmol/l and metabolic acidosis with Kussmaul breathing (pH = 6.80, HCO_3_ = 5 mmol/l, pCO_2_ = 23 mm Hg or 3.1 kPa). On admission to the ICU, she had a BP of 165/71 mm Hg, a HR of 85/min, an oxygen saturation of 98%, a body temperature of 36.8 °C, an arterial pO_2_ of 175 mm Hg (23.3 kPa), a base excess of −27 mmol/l, and an anion gap of 25 mmol/l. The initial lactic acid level was 16 mmol/l. Moreover, the patient displayed elevated serum ketoacids of 6.4 mmol/l and a BG of 76 mg/dl. Diabetes-related laboratory findings were indicative of type 2 diabetes: C‑peptide 2.34 ng/ml (0.78–1.89), insulin antibodies 1.0 U/ml (0.0–9.0), tyrosine phosphatase antibodies 0.0 U/ml (0.0–20.0), glutamic acid decarboxylase antibodies 2.2 U/l (0.0–9.5). Chest X‑ray on admission was normal. Hemodialysis and parenteral glucose infusion was started and metabolic acidosis thus receded over time (Fig. 1b). Renal function slowly recovered, the patient was switched to an insulin regimen and discharged after 14 days.

Patient 3: a 71-year-old man (BMI 30.0 kg/m^2^) on metformin, canagliflozin, liraglutide, enalapril, allopurinol, pantoprazole and torasemid presented to the ED because of acute gastroenteritis lasting for 6 days. The laboratory analyses revealed acute renal failure (creatinine 1203 µmol/l, blood urea nitrogen 36.9 mmol/l) and metabolic acidosis with hyperventilation (pH = 7.16, HCO_3_ = 10 mmol/l, pCO_2_ = 20 mm Hg or 2.7 kPa). On admission, the BP was 111/38 mm Hg, HR 73/min, oxygen saturation 98%, body temperature 35.8 °C, arterial pO_2_ 88 mm Hg (11.7 kPa), base excess −20 mmol/l, and the anion gap was 21.6 mmol/l. The initial lactic acid level was 5.9 mmol/l, serum ketoacids were ≥8 mmol/l at a blood glucose level of 150 mg/dl. Abdominal sonography and chest X‑ray revealed no pathologies. Continuous venovenous hemofiltration was started and parenteral glucose infusion initiated. Metabolic acidosis receded (Fig. 1c) and renal function slowly recovered. The patient was discharged after 9 days.

## Conclusion

To conclude, we report on three critically ill patients with MALKA with metformin accumulation due to acute renal failure. The concurrent occurrence of lactic acidosis and ketoacidosis was reflected in an augmented anion gap. The underlying mechanism contributing to ketoacidosis-aggravated metabolic acidosis could be starvation; however, ketoacid production for alternative energy during starvation usually induces mild metabolic acidosis [3]. Beta-hydroxybutyrate levels in starvation [3] undercut the levels measured in our patients. In the cases presented, metformin-induced inhibition of gluconeogenesis and stimulation of fatty acid oxidation is a likely co-factor for ketogenesis in the absence of other known ketogenic situations, such as pregnancy [4, 5] or alcohol intake [5].

To the best of our knowledge, in the studies published on MALA, ketoacids have not been systematically measured and the anion gap was explained solely by lactate and uremia. In support of our postulated hypothesis of suppressed hepatic gluconeogenesis in MALA leading to euglycemic ketoacidosis, a study in rats has shown the induction of ketoacid production by metformin treatment [2]. Likewise, post-mortem biochemistry analysis of beta-hydroxybutyrate and glucose of the previously mentioned fatal case with metformin overdosing [6] supports our findings.

The mechanism of metformin-associated ketogenesis seems to differ from lack of insulin in type 1 diabetes. Parenteral glucose terminates starvation ketoacidosis and counteracts the suppressed hepatic gluconeogenesis in MALKA. Accordingly, patients with MALKA seem to benefit primarily from parenteral glucose infusion, reflected in a case report of starvation metabolic acidosis [7] and the management of euglycemic ketoacidosis in pregnancy [4]. Interestingly, new evidence suggests that SGLT2-inhibitors might also cause euglycemic ketoacidosis [8]. As the third patient was on canagliflozin and metformin treatment, discrimination of the cause of euglycemic ketoacidosis, i.e. SGLT2 inhibitor and/or biguanide therapy, is currently impossible in that patient.

Taken together, our case series highlights the parallel occurrence of MALA and euglycemic ketoacidosis, the latter exceeding ketosis observed during starvation, suggesting a metformin-triggered inhibition of gluconeogenesis. The occurrence of MALA should prompt investigations for euglycemic ketoacidosis, and euglycemic ketoacidosis should lead to measurement of serum lactate levels in patients on metformin. Furthermore, prospective studies need to clarify the importance of early implementation of glucose infusions in cases of MALKA.

## Abbreviations

MALA: Metformin-associated lactic acidosis with concurrent euglycemic ketoacidosis
MALKA: Metformin-associated lactic acidosis with euglycemic ketoacidosis
CKD: Chronic kidney disease
ED: Emergency department
HR: Heart rate
SGLT2 inhibitor: Sodium-dependent glucose transporter 2 inhibitor
